# Quality of Life of Lower Limb Lymphatic Filariasis Patients in a North Indian Tertiary Care Center: A Prospective Observational Study

**DOI:** 10.7759/cureus.81920

**Published:** 2025-04-08

**Authors:** Sanjeev Kumar, Medhavi Gautam, Pankaj Singh, Shahrukh Rauf, Shiva S, Shubhajeet Roy, Suresh Kumar

**Affiliations:** 1 Department of Surgery, King George's Medical University, Lucknow, IND; 2 Department of Medicine, King George's Medical University, Lucknow, IND; 3 Department of General Surgery, King George's Medical University, Lucknow, IND; 4 Department of Surgical Gastroenterology, Sanjay Gandhi Postgraduate Institute of Medical Sciences, Lucknow, IND; 5 Department of Surgery, Gandhi Memorial and Associated Hospital, King George's Medical University, Lucknow, IND

**Keywords:** chronic disease, lymphatic filariasis, lymphatic filariasis specific quality-of-life questionnaire, lymphedema, neglected tropical diseases, quality of life

## Abstract

This observational study was carried out at a tertiary care facility in North India. A total of 154 patients with different degrees of lymphedema and clinically diagnosed lower limb lymphatic filariasis (LF; ages 15-75 years) were included. Seven dimensions of quality of life (QoL) were assessed using the Lymphatic Filariasis Specific Quality-of-life Questionnaire (LFSQQ; higher scores indicate a better overall QoL). There was a significant (p<0.001) difference in all the QoL domains among different grades. All QoL domains significantly worsened (p<0.001) among patients with disease grade III compared to grade I. However, pain/discomfort, psychological health, social participation, and total scores were significantly (p<0.001) lower among patients with disease grade III than II. The LFSQQ and overall QoL response were significantly different (p<0.001) among duration of disease groups and grades of disease. The study shows that LF has a significant negative effect on quality of life, especially in individuals who have a long-term illness and are in advanced stages of the disease.

## Introduction

Chronic lymphatic system dysfunction resulting in swelling of body tissues, usually in the limbs, is called lymphedema. Leprosy, lymphatic filariasis (LF), and podoconiosis are the main neglected tropical diseases (NTDs) that induce lymphedema in the tropics [1]. NTDs are mostly illnesses that affect underprivileged urban and rural populations, most of whom do not have access to basic medical care [2]. With improved sanitation efforts, efforts by the World Health Organization like the Global Programme to Eliminate Lymphatic Filariasis, and efforts by the Indian government like the National Vector Borne Disease Control Program, the incidence of LF has decreased, and other causes like surgery, radiation, and trauma, have become some of the more common causes of lymphedema currently [3].

The impact on daily routines, occupation status, and emotional, financial, and social issues are only a few of the elements that have been stated above that impact the quality of life (QoL) for LF sufferers. Patients with LF frequently experience acute painful episodes in the form of acute dermato-lymphangio-adenitis (ADLA) or acute filarial lymphangitis (AFL), which adds to the burden. In order to lessen impairment, the Global Programme to Eliminate Lymphatic Filariasis (GPELF) focuses on managing morbidity and preventing the spread of LF parasites [4,5]. 

The Institute of Applied Dermatology in Kerala, India, created the validated Lymphatic Filariasis Specific Quality-of-Life Questionnaire (LFSQQ) to measure the QoL of patients with LF [6]. The questionnaire covers a wide range of LF-related issues. The total score is a number between 0 and 100, where 0 represents the lowest QoL and 100 the highest; the higher the score, the better the QoL.

The aim of our prospective observational study was to assess the quality-of-life parameters among patients with lower limb LF in a North Indian population.

The abstract has been presented at the International Surgical Week (50th World Congress of Surgery), in Kuala Lumpur, Malaysia, in August 2024.

## Materials and methods

This was a prospective observational study conducted in the Department of General Surgery in a North Indian tertiary care center, from July 2021 to December 2022.

Patients aged 15-75 years with lower limb lymphatic filariasis (diagnosed by the diethylcarbamazine (DEC) provocation test, DEC is administered orally in small doses of 2 mg/kg. After 30 to 45 minutes, a blood sample is obtained to look for microfilariae. By stimulating microfilariae and detecting them in peripheral blood, the DEC provocative test, which is used to diagnose lymphatic filariasis, enables daytime detection rather than relying on nocturnal blood samples), with Grade I, II, or III lymphedema as per Brunner’s Classification (Grade I (Reversible): There is pitting edema, but it resolves on lower limb elevation. Grade II (Spontaneously Irreversible): Tissue fibrosis may develop, causing the afflicted limb to thicken and stiffen, and swelling is non-pitting and does not go away with elevation. Grade III (Elephantiasis): extreme swelling, skin abnormalities like thickening and wart-like growths, and the possibility of lymphostatic elephantiasis (large, malformed limb) are the hallmarks of this most severe stage.), and a clinical and microbiological diagnosis of lymphatic filariasis were included in the study. Patients with acute attacks of acute adenolymphangitis and hydrocele were excluded from the study.

Data collection

The patients who consented to participate in the study were asked to fill out the LFSQQ Questionnaire, which was administered by the investigators. The LFSQQ questionnaire covers a range of lymphatic filariasis-related problem categories. Using 44 questions, under seven domains, self-care, mobility, regular activities, illness burden, pain/discomfort, psychological health, and social participation, it evaluates the patients' health state over the previous thirty days. There are several questions and items in each domain that address issues related to the condition of the disease. There are five categories in which each item is scored: no problem (4 scores), mild (3 scores), moderate (2 scores), severe (1 score), and most severe (0 scores). Although not all questions might be applicable to all patients, the maximum and minimum possible scores are 176 and 0, respectively. The overall QoL response is determined using the total number of questions answered and the raw score, accounting for questions that may not be applicable to all patients. The total score and answer in terms of quality of life are computed by:

Overall QoL response = Total score / (4 x number of questions answered) x 100

Overall QoL response ranges from 0 to 100, where zero indicates the worst QoL and 100 indicates the best possible QoL, i.e., the higher the score, the better is the quality of life.

Statistical analysis

The findings are displayed as mean±SD, frequencies, and percentages. The quality of domains among various disease grades and duration groups was compared using one-way ANOVA and Tukey's post hoc test. A significant p-value was defined as less than 0.05. The SPSS v24.0 (IBM, Chicago, Inc., USA) was used for all of the analysis.

Ethics

The Institutional Ethics Committee of King George’s Medical University, Lucknow approved the study (ECR/262/Inst/UP/2013/RR-19), and prior to participant enrolment, each subject's informed consent was obtained.

## Results

A total (n) of 154 patients were included in our study, whose average age was 41.27±14.71 years (range: 15-75 years). About one-fourth of the patients were between 41 and 50 years (23.4%). More than half of the patients were males (63.6%) (Table 1).

**Table 1:** Age and gender distribution of the patients. The mean and range of ages for the patients were 41.27±14.71 years (15-75 years).

Age and gender	No. (n=154)	%
Age in years		
≤20	15	9.7
21-30	29	18.8
31-40	33	21.4
41-50	36	23.4
51-60	27	17.5
>60	14	9.1
Gender		
Male	98	63.6
Female	56	36.4

The scores of different domains of QoL have been summarized in Table 2.

**Table 2:** Scores of various domains of quality of life (LFSQQ questionnaire). LFSQQ: Lymphatic Filariasis Specific Quality-of-life Questionnaire.

Domains	Mean±SD	Median
Mobility	17.83±9.25	20.00
Self-care	12.44±4.74	14.00
Usual activities	13.82±7.53	15.00
Disease burden	12.77±4.63	14.00
Pain/discomfort	16.69±6.65	18.00
Psychological health	17.30±7.09	18.00
Social participation	11.95±5.63	13.50
Total score	100.98±40.26	113.00

The total score was 100.98±40.26 with a median of 113.00.

There was a significant (p<0.001) difference between the different grades of the disease in all the quality-of-life domains’ scores. As per post-hoc analysis, all the quality-of-life domains’ scores were significantly (p<0.001) lower in the patients with disease grade III, compared to those with grade I. However, pain/discomfort, psychological health, social participation, and total scores were significantly (p<0.001) lower among patients with disease grade III than those with grade II (Table 3).

**Table 3:** Comparison of different quality of life domains (LFSQQ) scores across various disease grades. Note: ^1^ANOVA test used; '*' indicates significance. 'a' denotes a significant correlation between Grades I and III, and 'b' denotes a significant correlation between Grades II and III, both with p<0.001 (post-hoc tests). LFSQQ: Lymphatic Filariasis Specific Quality-of-life Questionnaire.

Domains	Grade of disease			P-value^1^
	Grade I (n=47)	Grade II (n=61)	Grade III (n=46)	
	Mean±SD	Mean±SD	Mean±SD	
Mobility	25.13±4.61^a^	20.80±6.23	6.74±4.69^a^	<0.001*
Self-care	15.96±2.14^a^	13.87±3.21	7.09±3.53	<0.001*
Usual activities	19.23±5.49^a^	16.18±5.28	5.38±3.55	<0.001*
Disease burden	16.77±2.84^a^	13.65±2.65	7.66±3.18	<0.001*
Pain/discomfort	22.45±4.03^a^	18.10±3.69^b^	9.15±4.35	<0.001*
Psychological health	22.83±4.50^a^	19.03±4.54^b^	9.55±4.88	<0.001*
Social participation	16.68±2.28^a^	13.52±3.29^b^	5.23±3.77 ^a,b^	<0.001*
Total score	139.04±13.68^a^	115.15±17.57^b^	50.81±17.30^a,b^	<0.001*

There was a significant (p<0.001) difference in self-care, pain/discomfort, social participation, and total scores among groups of different durations of the disease. The post-hoc tests revealed that the self-care score was significantly (p<0.001) lower among patients whose duration of disease was ≥10 years (9.09±6.12), than those of 1-<5 years (17.83±6.00). Pain/discomfort, social participation, and total score were significantly (p<0.001) lower among patients whose duration of disease was ≥10 years, than those of <1 year. It was also observed that the total score was significantly (p<0.001) lower among patients whose duration of disease was ≥10 years (68.36±36.41), than those of 1-<5 years (123.46±26.76) (Table 4).

**Table 4:** Comparison of different quality of life domains' (LFSQQ) scores with duration of disease. Note: ^1^ANOVA test used; '*' indicates significance. 'a' and 'b' denote p<0.001 (post-hoc tests), where 'a' signifies a significant difference between the 1st and 4th columns, and 'b' signifies the same between the 2nd and 4th columns. LFSQQ: Lymphatic Filariasis Specific Quality-of-life Questionnaire.

Domains	Duration of disease				^1^p-value
	<1 years (n=36)	1-<5 years (n=41)	5-<10 years (n=24)	≥10 years (n=53)	
	Mean±SD	Mean±SD	Mean±SD	Mean±SD	
Mobility	14.75±4.08	14.80±3.66	11.33±4.51	9.53±4.29	<0.001*
Self-care	16.42±7.59	17.83±6.00^b^	13.50±7.17	9.09±6.12^b^	<0.001*
Usual activities	14.83±4.90	14.71±3.19	11.29±4.32	10.55±4.33	<0.001*
Disease burden	19.86±6.53	19.83±4.83	14.25±5.20	13.23±6.46	<0.001*
Pain/discomfort	21.39±6.11^a^	19.76±5.95	14.63±6.53	13.83±6.68^a^	<0.001*
Psychological health	14.81±4.71	14.37±3.87	10.50±4.72	8.81±5.97	<0.001*
Social participation	16.25±7.65^a^	12.45±6.14	11.25±5.80	5.60±3.01^a^	<0.001*
Total score	126.97±32.0^a^	123.46±26.76^b^	92.17±36.45	68.36±36.41^a,b^	<0.001*

The LFSQQ scores ranged from 20.0 to 161.0. The mean value of the LFSQQ was 100.98±40.26. The mean value of the overall quality-of-life response (%) was 66.24±25.20 (Table 5).

**Table 5:** Lymphatic filariasis-specific quality of life questionnaire (LFSQQ) and overall quality of life response of the patients.

	Mean±SD	Median
LFSQQ score	100.98±40.26	112.00
Overall quality-of-life response (%)	66.24±25.20	74.00

The LFSQQ score and the overall quality-of-life response were significantly (p<0.001) different among different duration of disease groups and grades of disease. The post hoc tests revealed that the LFSQQ score and the overall quality-of-life response were significantly (p<0.001) lower among patients whose duration of disease was ≥10 years than those of <1 year. The LFSQQ scores and the overall quality-of-life response were also significantly (p<0.001) lower among patients with disease grade III than those with grade I (Table 6).

**Table 6:** Comparisons of lymphatic filariasis-specific quality of life questionnaire (LFSQQ) scores and overall quality of life response of patients by duration and grades of disease. Note: ^1^ANOVA test used; '*' indicates significance. 'a' denotes a significant difference in LFSQQ scores among various disease duration groups; 'b' denotes a significant difference in overall quality of life scores among various disease duration groups; 'c' denotes a significant difference in LFSQQ scores among various disease grade groups; and 'd' denotes a significant difference in overall quality of life scores among various disease grade groups.

Duration and grades of disease	LFSQQ score	P-value^1^ (LFSQQ Score)	Overall quality-of-life score (%)	P-value^1^ (Overall quality-of-life score)
Duration of disease		<0.001*		<0.001*
<1 years (n=36)	124.86±32.16^a^		82.27±20.19^b^	
1-<5 years (n=41)	122.51±26.66		79.32±16.57	
5-<10 years (n=24)	90.54±36.94		60.85±23.80	
≥10 years (n=53)	72.83±36.11^a^		47.67±21.31^b^	
Grade of disease		<0.001*		<0.001*
Grade I (47)	137.74±1457^c^		89.33±8.18^d^	
Grade II (61)	112.36±21.68		74.24±10.76	
Grade III (46)	48.33±15.66^c^		32.03±9.80^d^	

## Discussion

Using the LFSQQ, we questioned each patient to determine their QoL while conducting our study. Selected patients with varying disease grades were included in the assessment. The QoL of patients having the disease for a longer duration with highly progressed grade III disease was affected in all seven domains (p<0.001). The overall LFSQQ score and the overall QoL response (%) were significantly lower in those with ≥10 years of disease duration or with higher grades of disease (p<0.001). The disfiguring nature of the disease led to disturbed psychosocial health in the patients. The affected patients had decreased social participation and mainly resorted to staying at home, leaving their work, which in turn increased the burden on their caretakers. The affected females, who were mostly housewives, faced problems in carrying out normal household chores like washing clothes, cleaning floors, etc. In a previous study on depressive illness due to the disease, it was concluded that the stigma associated with the physical appearance due to the disease induced mental illness [7].

A few other studies have described the impact of LF on mobility and acute attack issues [8, 9, 10]. According to research on lymphedema patients' health-related QoL, these individuals have more psychological issues than the general population [11]. An Indian study found that individuals with lymphedema experienced difficulties with movement (67-78%), economic activities (65-83%), and household tasks (15-33% of patients) [12]. According to these studies, the majority of patients reported that their illness made them feel alone, frustrated, or embarrassed [13, 14]. The negative impact on mobility and usual daily activities, mostly in grade III disease (p<0.001), affected the economic productivity of patients. The self-care domain was also affected, which further deteriorated limb swelling and predisposed the patients to secondary infections.

In a separate study, the LFSQQ was employed to assess the impact of LF cases in nine communities on the QoL of those affected. Grade II lymphedema was seen in 25.8% of cases. The overall average QoL score was 68.24. The quality of life and the lymphedema stage had a significant negative correlation (p<0.001). Additionally, there was a significant positive correlation (p<0.001) between the pain/discomfort score and the illness load. The environmental domain had the lowest domain-specific score (45.94), whereas self-care had the highest score (85.03) [15].

A prior study that used the WHO 100-item QoL questionnaire (WHOQOL-100) on 141 patients with filarial lymphedema in Sri Lanka found that, in comparison to patients with lymphedema, healthy controls had a higher overall general quality of life and performed better in all domains [16]. According to another study, LF also impacts the economic activities of the productive age group, leading them to reduce their working hours, switch employment, or quit altogether [13].

It is often known that the physical abilities of LF participants are impaired compared to those of healthy individuals who do not have the illness [17-19]. However, other studies have indicated that LF patients practice self-care at a higher level [20, 21]. In evaluating the patients' physical skills as well as the different illness symptoms (adenolymphangitis (ADL), hydrocele, lymphedema, and elephantiasis), the mobility of LF patients is also seen to be crucial. The employment and income status of those affected by the ailment are negatively impacted by this [14, 22].

The significant effects of LF on patients' psychological and financial well-being, especially in those with advanced disease stages, are still highlighted in recent research. More than 70% of patients with stage III lymphedema had significant difficulties carrying out both instrumental and basic activities of daily living, according to a 2023 multicentric survey conducted in Southeast Asia. This frequently led to long-term unemployment and decreased household income [23]. This is consistent with our findings that self-care and social participation were significantly impacted by disease durations of ≥10 years. Due to gender roles in household chores, women were disproportionately affected by the stigma and social disengagement experienced by LF patients with chronic sequelae, including recurring ADL and skin infections, according to a 2022 systematic study [24]. Nearly 50% of people with chronic illnesses had mental health comorbidities, especially anxiety and depression, which suggests the need for integrated psychosocial therapies [25]. Additionally, following suggested self-care routines improved QoL scores over a six-month period, particularly in the domains of mobility and pain/discomfort, according to a 2023 field evaluation of morbidity management and disability prevention (MMDP) programs [26]. In order to lessen the long-term burden of LF, these new findings highlight the significance of early intervention, community-based education, and reliable access to resources for morbidity control.

Limitations

First, the results may not be broadly applicable to other areas or populations due to the small sample size and the restriction to a single tertiary care facility in North India. Second, because the study was observational in nature, a causal relationship between LF and the noted quality-of-life outcomes cannot be proven. Third, patients may overreport or underreport their symptoms and difficulties if the LFSQQ is used to collect self-reported data, which could lead to response bias. Additionally, the total illness burden may have been underestimated due to the omission of patients who had acute episodes of hydrocele and adenolymphangitis. Lastly, the study's cross-sectional design records a single moment in time and ignores possible variations in the severity of the illness or QoL over time. These results could be validated and expanded upon by larger, more diverse population studies in the future using longitudinal methods. Additionally, the absence of a healthy control group makes it difficult to definitively attribute the observed QoL deficits solely to LF, as other factors might also contribute.

## Conclusions

Our research emphasizes, through the administration of the LFSQQ, that patients with higher disease grades and longer disease durations demonstrate significantly worse QoL ratings in a number of different domains. The disease's deformity and chronic nature cause severe psychological and financial difficulties, which decrease social interaction and increase reliance on caregivers. These results highlight the significance of comprehensive management approaches that address the psychological and financial needs of afflicted individuals in addition to the medical treatment of LF. Our findings underscore the necessity of focused therapies to enhance these patients' QoL.
